# Supplementary material for: Splice-Junction-Based Mapping of Alternative Isoforms in the Human Proteome
Source: Cell Rep. Author manuscript; Available in PMC 2020 Jan 15. (PMC6961840; doi:10.1016/j.celrep.2019.11.026)
Supplement: 2 [file NIHMS1546469-supplement-2.zip › DF1/PXD009737/TestisTrypsin/TestisTrypsin_74_KIF21B_QLALGNVISALGDQSK.pdf]

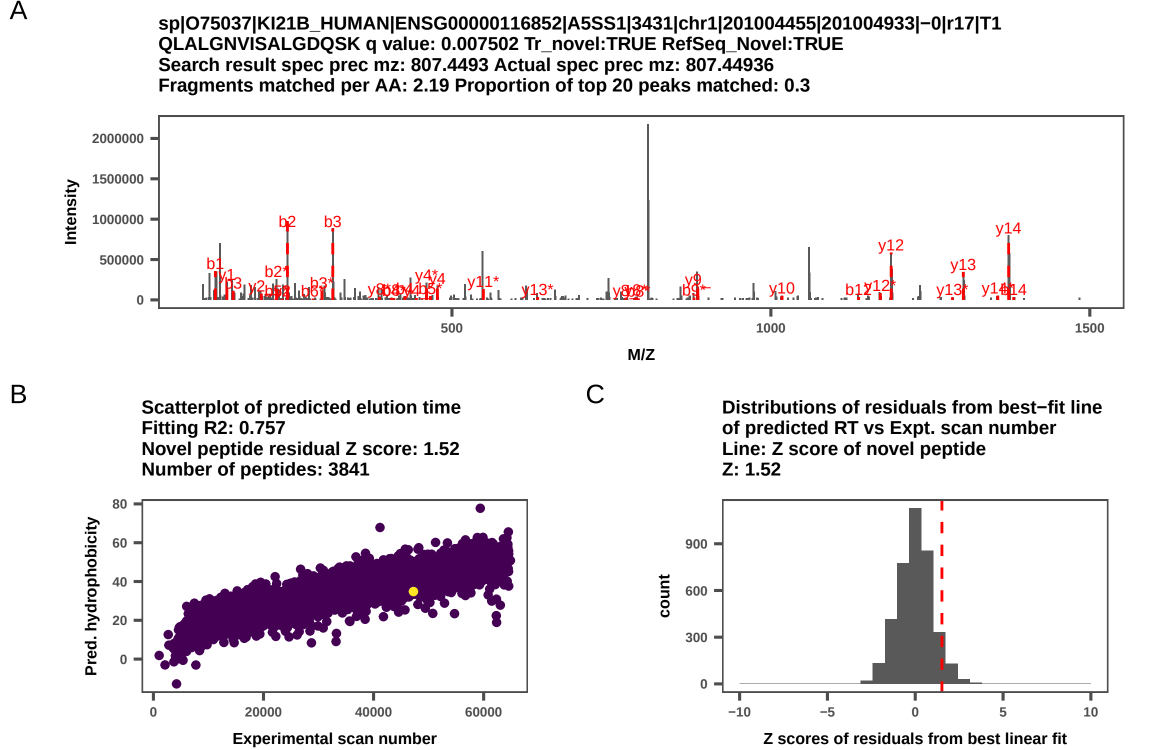
A
sp|O75037|KI21B_HUMAN|ENSG00000116852|A5SS1|3431|chr1|201004455|201004933|−0|r17|T1
QLALGNVISALGDQSK q value: 0.007502 Tr_novel:TRUE RefSeq_Novel:TRUE
Search result spec prec mz: 807.4493 Actual spec prec mz: 807.44936
Fragments matched per AA: 2.19 Proportion of top 20 peaks matched: 0.3
Intensity
2000000
1500000
1000000
500000
0
b1
b2
b3
b2*
b3*
y4*
y4
y11*
y13*
y9
b9*
y10
b12
y12
y12*
y13
y13*
y14
M/Z
500
1000
1500
B
Scatterplot of predicted elution time
Fitting R2: 0.757
Novel peptide residual Z score: 1.52
Number of peptides: 3841
Pred. hydrophobicity
80
60
40
20
0
0
20000
40000
60000
Experimental scan number
C
Distributions of residuals from best−fit line
of predicted RT vs Expt. scan number
Line: Z score of novel peptide
Z: 1.52
count
900
600
300
0
−10
−5
0
5
10
Z scores of residuals from best linear fit
